# The importance of multiprofessional care

**DOI:** 10.1590/1516-3180.2021.139223022021

**Published:** 2021-04-05

**Authors:** Paulo Manuel Pêgo Fernandes, Gabriela Favaro Faria

**Affiliations:** I PhD. Full Professor, Thoracic Surgery Program, Instituto do Coracao, Hospital das Clinicas HCFMUSP, Faculdade de Medicina, Universidade de Sao Paulo, Sao Paulo, SP, BR.; II Assistant Director, Discipline of Thoracic Surgery, Instituto do Coracao, Hospital das Clinicas HCFMUSP, Faculdade de Medicina, Universidade de Sao Paulo, Sao Paulo, SP, BR.

Multiprofessional care can be defined as a work methodology involving healthcare professionals “with complementary backgrounds and skills, sharing common health goals and exercising concerted physical and mental effort in assessing, planning, or evaluating patient care. This is accomplished through interdependent collaboration, open communication and shared decision-making. This in turn generates value-added patient, organizational and staff outcomes.”^1^

Within the context of Brazilian governmental healthcare actions, this care model is recent. It began through changes in public healthcare policies that led to creation of the National Health System (SUS). SUS took on the challenge of replacing the existing care practices that focused on curing diseases. It brought in plans and strategies aimed towards the principles of universality, equity and comprehensiveness of care. In 1994, the Family Health Program (PSF) was created, which was directed towards the context of primary healthcare. The strategy for attending the population was developed around multiprofessional work that had the aim of developing educative activities focusing on problem-solving and transformation of realities.^2^^,^^3^

The teams are generally composed of doctors, nurses, dentists, oral health technicians or auxiliaries, nursing technicians or auxiliaries and community health agents (ACS). The main objectives are to promote autonomy and stimulate self-care, thus seeking better quality of life for individuals and communities, while respecting the realities and the environment that surround these individuals.^4^^,^^5^

One of the biggest difficulties in implementing this is the lack of professionals to meet this demand in all the spheres of public healthcare. To overcome this, a change in culture and commitment towards public administration is fundamental, in order to achieve practices governed by preventive actions and health promotion.^6^

One example of multiprofessional care is the palliative care that traditionally forms the therapeutic option for end-stage oncological patients. Within this scenario, multiprofessional teams have an essential role in symptom alleviation, improvement of quality of life and improvement of comfort for patients and their families.^7^

Palliative care teams are frequently composed of doctors, nurses, social assistants, volunteers and religious leaders. Whenever possible, patients should be the ones to make final decisions on their own care, using information from the team and their own values as a guide.^8^

Within all spheres of patient care, teamwork provides direct and indirect improvements for everyone involved in the process. These include reduction of hospital length of stay, shortening of recovery time and improvement of adherence to treatment.^9^ A systematic review in the literature examined the actions of multidisciplinary teams at different stages of oncological treatment (diagnosis, treatment, pain control and palliative care). In all the studies analyzed, there was a multidisciplinary team and a control group. The multidisciplinary team improved adherence to treatment and diminished the time taken to do tests, thus enabling higher chances of cure. Discussion of cases among the team members had a positive impact on planning and implementing therapies, making clinical decisions and making referrals to specialists. Within palliative care, there was better pain control and higher adherence to oral medications. The study showed that formation of multidisciplinary teams to act in relation to cancer treatment is a promising development that can improve patients’ quality of life and the efficiency of the services provided.^10^

In relation to transplantation, multiprofessional teams are essential at all stages of the process. For organ donation, synchronicity of activities is fundamental given that the tasks of each member of the team are complementary and are all of prime importance.^11^

Some examples of the activities of multiprofessional teams relating to organ donation: The nurse does the active search, notifies the transplantation center, communicates with the medical team, conducts interviews with the family, collects samples for laboratory tests and operates the extracorporeal circulation machine. **The doctor** conducts the protocols for certifying brain death, assesses the viability of the organs and harvests the organs. **The psychologist** provides emotional support for the donor's family and conducts interviews with the family. **The social assistant** aids the family in signing documentation for the donation and provides guidance for the family if the body needs to be moved.

It has now been seen that multiprofessional teams have made great efforts towards combating the pandemic of severe acute respiratory syndrome coronavirus 2 (SARS-CoV-2). In facing up to its disease (COVID-19), development of research and care protocols and dissemination of information have certainly been a challenge.^12^

At the Heart Institute (Instituto do Coração, INCOR) of Hospital das Clínicas, University of São Paulo Medical School (Faculdade de Medicina da Universidade de São Paulo, FMUSP), a multiprofessional committee for managing the COVID-19 crisis was set up. This group has been holding weekly meetings with the executive, clinical and nursing directorates and with the hospital infection committee, in order to discuss and pass on information to the different professionals, regarding new measures at the institution for facing the pandemic, and to draw up care protocols and training schemes to capacitate professionals to care for these patients.

In providing care for patients with COVID-19, pulmonologists, cardiologists, intensive care specialists, nurses, physiotherapists, pharmacists, nutritionists and psychologists together define therapeutic plans for these patients, starting from individual assessments on each case. Actions are implemented in accordance with the priorities and targets that have been established.

Even if there is a favorable outcome after hospitalization, patients with other diseases of high complexity who then go through the COVID-19 experience and require intensive care are generally left with a significant physical and psychological burden. In this regard, multiprofessional teams need to be organized such that they can provide long-term care until these patients’ health, or at least their quality of life, is restored.^13^

Multiprofessional care is a recently proposed way of working that has become widely used among healthcare teams in order to face up to the intense process of specialization and fragmentation of care. Formation of teams focusing on meeting needs comprehensively and on seeking solutions that complement each other in an effective manner is a strategy that enables care that is safer and provided by professionals with better qualifications. Moreover, this brings better results for patients.
